# Pathophysiology of Motor Dysfunction in Parkinson's Disease as the Rationale for Drug Treatment and Rehabilitation

**DOI:** 10.1155/2016/9832839

**Published:** 2016-06-06

**Authors:** Francesca Magrinelli, Alessandro Picelli, Pierluigi Tocco, Angela Federico, Laura Roncari, Nicola Smania, Giampietro Zanette, Stefano Tamburin

**Affiliations:** ^1^Department of Neurosciences, Biomedicine and Movement Sciences, University of Verona, Piazzale Scuro 10, 37134 Verona, Italy; ^2^Neuromotor and Cognitive Rehabilitation Research Centre, University of Verona, Piazzale Scuro 10, 37134 Verona, Italy; ^3^Rehabilitation Unit, Pederzoli Hospital, Via Monte Baldo 24, 37019 Peschiera del Garda, Italy; ^4^Neurology Unit, Pederzoli Hospital, Via Monte Baldo 24, 37019 Peschiera del Garda, Italy

## Abstract

Cardinal motor features of Parkinson's disease (PD) include bradykinesia, rest tremor, and rigidity, which appear in the early stages of the disease and largely depend on dopaminergic nigrostriatal denervation. Intermediate and advanced PD stages are characterized by motor fluctuations and dyskinesia, which depend on complex mechanisms secondary to severe nigrostriatal loss and to the problems related to oral levodopa absorption, and motor and nonmotor symptoms and signs that are secondary to marked dopaminergic loss and multisystem neurodegeneration with damage to nondopaminergic pathways. Nondopaminergic dysfunction results in motor problems, including posture, balance and gait disturbances, and fatigue, and nonmotor problems, encompassing depression, apathy, cognitive impairment, sleep disturbances, pain, and autonomic dysfunction. There are a number of symptomatic drugs for PD motor signs, but the pharmacological resources for nonmotor signs and symptoms are limited, and rehabilitation may contribute to their treatment. The present review will focus on classical notions and recent insights into the neuropathology, neuropharmacology, and neurophysiology of motor dysfunction of PD. These pieces of information represent the basis for the pharmacological, neurosurgical, and rehabilitative approaches to PD.

## 1. Introduction

Parkinson's disease (PD) is the second most common neurodegenerative disorder after Alzheimer's disease (AD), with an overall prevalence of 300 per 100,000 [1] that rises from 41 in the 40–49 years' age range to 1903 in people older than age of 80 years [2].

PD has been traditionally considered as a pure movement disorder secondary to focal degeneration of dopaminergic neurons in the substantia nigra, but, in recent years, the clinical phenotype has been better illuminated, showing that PD is a multisystem neurodegenerative disorder with motor and nonmotor features (Table 1) [3]. Among motor symptoms and signs, the cardinal ones (bradykinesia, rest tremor, and rigidity) are mainly ascribed to the loss of dopaminergic neurons [4], but those involving posture, balance, and gait are largely secondary to degeneration of nondopaminergic pathways and significantly contribute to impairment and disability in advanced PD patients [5]. Nonmotor features result from multiple neurotransmitter deficiencies in the central and peripheral nervous system [6] and include psychiatric (depression, apathy, hallucinations, and delusions) and autonomic (constipation, orthostatic hypotension, and urinary and genital disturbances) features, cognitive impairment (involvement of executive functions, memory, and visuospatial functions up to dementia) [7, 8], sleep disorders, olfactory dysfunction, and pain [9] that together contribute to worsening the quality of life (QoL) and patient's disability [6].

Multiple agents have been studied in randomized controlled trials (RCTs) designed to assess disease modification or neuroprotection in PD, but all have failed [10], and medical treatment remains symptomatic [10]. Pharmacological therapy is based on levodopa and dopamine agonists and is very successful in the early stages of the disease, when dopaminergic symptoms and signs are predominant and long term motor complications still have not developed, but other treatment strategies are almost invariably necessary as time passes [3]. Long term levodopa-induced motor complications include motor fluctuations and dyskinesia and affect almost all PD patients at some point during the disease course, with relevant implications in global health status [11]. Despite various pharmacological approaches, as well as more invasive strategies including devices and functional neurosurgery, being available to manage such complications, many patients remain significantly disabled, and a fully satisfying management of motor complications is still an unmet need of PD therapy [11]. Nonmotor symptoms and signs are integral to PD at onset and throughout the disease course, but to date their treatment is largely unsatisfactory [9].

This review will summarize the evidence on the pathophysiology of PD motor symptoms and signs and give some insight into their neuropathological and neuropharmacological bases. These pieces of information may help the clinicians to better understand the rationale of current pharmacological and rehabilitation strategies for PD and encompass the large areas of uncertainty that should represent the focus for further studies.

## 2. The Functional Anatomy and Pathophysiology of the Basal Ganglia and the Role of the Cerebellum

**Figure 1:**
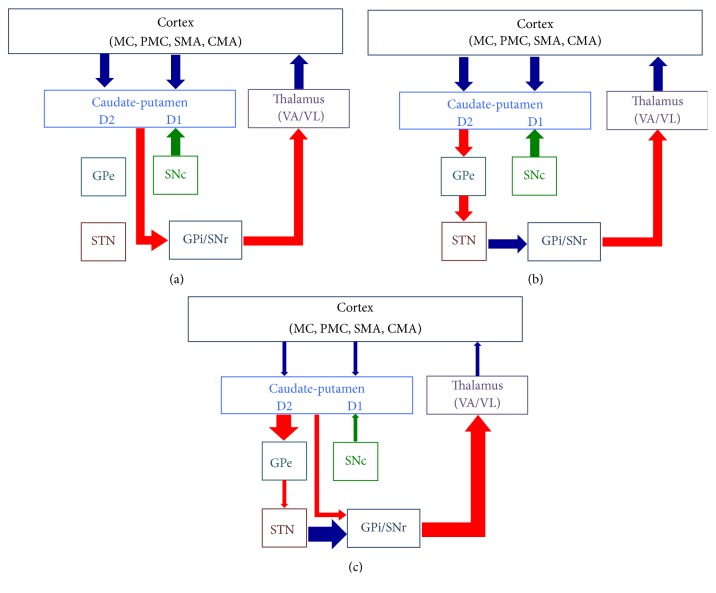
A simplified view of the functional anatomy of the basal ganglia (BG). The main input and output connections and the basic internal circuitry of the BG are shown. Here are represented the direct pathway (panel (a)), the indirect pathway (panel (b)), and the alteration of the balance between the direct and indirect pathways in Parkinson's disease (panel (c)). Blue arrows show the excitatory glutamatergic pathways, red arrows indicate the inhibitory GABAergic pathways, and green arrows mark the dopaminergic pathway. CMA: cingulate motor area; D1: dopamine D1 receptor; D2: dopamine D2 receptor; GPe: external segment of the globus pallidus; GPi: internal segment of the globus pallidus; MC: primary motor cortex; PMC: premotor cortex; SMA: supplementary motor area; SNc: substantia nigra pars compacta; SNr: substantia nigra pars reticulata; STN: subthalamic nucleus; VA/VL: ventral anterior/ventrolateral thalamic nuclei.

**Figure 2:**
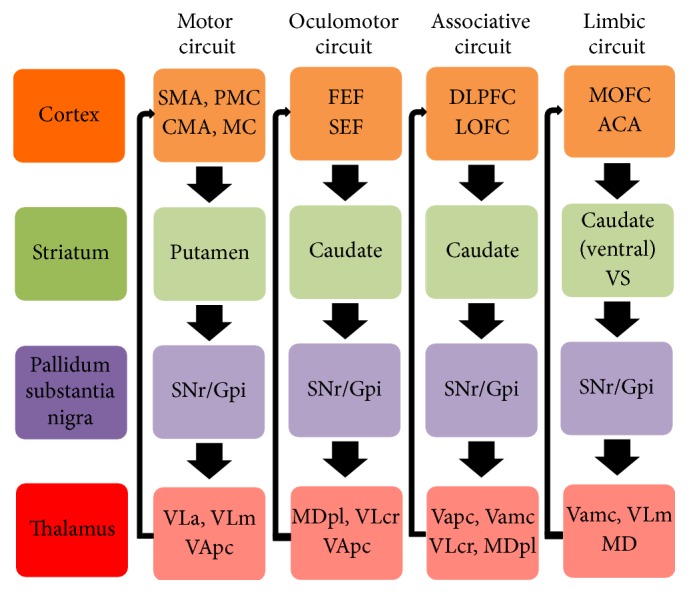
The parallel motor, oculomotor, associative, and limbic circuits of the basal ganglia. ACA: anterior cingulate area; CMA: cingulate motor area; DLPFC: dorsolateral prefrontal cortex; FEF: frontal eye fields; GPi: internal segment of the globus pallidus; LOFC: lateral orbitofrontal cortex; MC: primary motor cortex; MD: mediodorsal nucleus of the thalamus; MDpl: mediodorsal nucleus of thalamus, pars lateralis; MOFC: medial orbitofrontal cortex; PMC: premotor cortex; SEF: supplementary eye field; SMA: supplementary motor area; SNr: substantia nigra pars reticulata; VAmc: ventral anterior nucleus of thalamus, pars magnocellularis; VApc: ventral anterior nucleus of thalamus, pars parvocellularis; VLa: anterior ventrolateral nucleus of the thalamus; VLcr: ventrolateral nucleus of thalamus, pars caudalis, rostral division; VLm: ventrolateral nucleus of thalamus, pars medialis; VS: ventral striatum [12, 13].

The basal ganglia (BG) include the striatum, which comprises the caudate nucleus, putamen, and nucleus accumbens, the globus pallidus that is divided into an external segment (GPe) and an internal segment (GPi), the substantia nigra that can be divided into a pars compacta (SNc) and a pars reticulata (SNr), and the subthalamic nucleus (STN) [14]. The main input region of the BG is the striatum, which receives afferents from many regions of the cerebral cortex, including motor and premotor, cingulate, and prefrontal cortices, and the intralaminar nuclei of the thalamus [14–16]. The major output regions of the BG are the GPi and the SNr, which project to the thalamus modulating activity of cortical regions and to the brainstem [14–16]. The input and output regions are connected via either the direct or the indirect pathways, both of which arise from the matrix medium spiny neurons of the striatum (Figure 1), while the striosomal medium spiny neurons control dopaminergic projections from the SNc [14–17]. Corticostriatal projections, intrinsic BG circuits, and output pathways are functionally arranged according to the BG loop involved (Figure 2) [16, 17]. The main neurotransmitter of BG circuit is the inhibitory gamma-aminobutyric acid (GABA), while neurons of the STN use excitatory glutamate, and those of the SNc use dopamine [18]. Despite its oversimplification, the basic BG circuitry and the balance between the direct and indirect striatal pathways provide a simple heuristic model for PD cardinal signs and dyskinesia [16, 17]. According to this model, the pathophysiological hallmark of PD hypokinetic signs is the prevalence of the indirect pathway over the direct one resulting in increased neuronal firing activity in the output nuclei of the BG and leading to excessive inhibition of thalamocortical and brainstem motor systems, interfering with normal speed of movement onset and execution (Figure 1) [14–16]. At variance, overactivity in the direct pathway and imbalance with the indirect one may cause reduced inhibitory BG output and result in reduced BG filtering and parallel facilitation of multiple movement fragments causing dyskinesia, including those induced by levodopa in advanced PD [16, 19]. This model and its prediction of increased STN and GPi activity in PD fit well with the efficacy of targeting and inhibiting these two nuclei with deep brain stimulation (DBS), which represents the gold standard treatment of motor fluctuations and dyskinesia in advanced PD [20]. Despite its merits, this model is blinded to a number of experimental and clinical data, including the following issues: (a) the large number of BG neurotransmitters, neuromodulators, and their receptors goes beyond GABA, glutamate, and dopamine [21], and the complex arrangement of medium spiny neurons in matrix and striosome [17] does not fit well with a simple direct-indirect pathway imbalance; (b) the model should go beyond the simple concept of firing rate and include firing pattern, synchronization, and coincidence to better understand BG circuitry functioning; (c) while the model can convincingly explain bradykinesia, it fails to completely account for the appearance of rigidity and tremor; (d) pallidotomy or GPi DBS does not cause hyperkinesia, as predicted by this model, but may paradoxically reduce PD hyperkinetic signs; (e) hypokinetic and hyperkinetic signs can coexist in PD patients and cannot be simply considered as two sides of the same coin; (f) BG surgery and DBS can be performed with little or no apparent deficits [16, 22]. Future updated models of BG functions should incorporate a more complex BG circuitry and include nonlinear dynamics to address these issues [16].

The BG circuitries play a key role in selecting a motor program and inhibiting undesired ones and in movement preparation and execution, but their functions go beyond the motor system and include crucial functions such as learning, planning, executive functions, and emotions [14]. According to their connections, BG loops are functionally subdivided into motor, oculomotor, associative, and limbic ones (Figure 2) [12, 13, 16]. The motor loop is organized somatotopically and according to specific tasks or parts of a motor sequence [12, 16]. Abnormally synchronized oscillatory activity in this loop correlates with motor deficit in PD, and its suppression by dopaminergic therapies, ablative surgery, or DBS might provide the basic mechanism for the amelioration of motor impairment [23]. The oculomotor circuit is involved in the control of saccadic and smooth pursuit eye movements, which are abnormal in most PD patients [24]. The main abnormality consists of saccade hypometria, although all types (predictive, anticipatory, and memory-guided) of saccade generation may be involved [24]. Dopaminergic therapy and DBS of the STN reduce saccade latencies in parallel with the improvement of hand bradykinesia [25]. The dysfunction of the limbic circuit contributes broadly to some PD behavioral aspects, which include reward dysregulation phenomena, emotional blunting [26], and impulse control disorders secondary to dopaminergic treatment [27]. The associative loop takes part in prefrontal cognitive functions, and its impairment is responsible for cognitive inertia and executive dysfunction in PD [26].

The BG and the cerebellum modulate the activity of largely overlapping cerebral cortical areas through multisynaptic loops, which were traditionally assumed to be anatomically and functionally separate [28]. Recent studies showed that the dentate nucleus of the cerebellum projects to the striatum and to the GPe and that the STN has topographical projections to the cerebellar cortex via the pontine nuclei [29]. These reciprocal connections between the BG and the cerebellum, together with neuropathological changes in the cerebellum, account for the hypothesis that the cerebellum plays a role in the pathogenesis of PD symptoms and signs [28]. Functional MRI studies showed hyperactivation or strengthened connectivity in the cerebellum of PD patients [30], but whether it represents a pathogenetic or compensatory change is still debated [31]. Converging pieces of evidence accumulated recently in favor of a role of the cerebellum in some PD symptoms and signs, including tremor [32], gait disturbances through its connections with the pedunculopontine nucleus (PPN) [33], dyskinesia [34], and nonmotor symptoms, suggesting that the cerebellum might represent a promising new target for neuromodulation [28].

## 3. The Neuropathology of PD

The classical pathologic substrate for PD is the accumulation of neuronal inclusions composed of *α*-synuclein and called Lewy bodies and neurites and neuronal loss [35]. Neuronal loss is most marked in the SNc [35], but Lewy bodies in PD extend well beyond this region [36]. Based on the distribution of *α*-synuclein pathology, a staging scheme for PD has been proposed [36]. According to this scheme, neuronal pathology occurs early in the dorsal motor nucleus of the vagus and the olfactory bulbs, then spreads to the locus coeruleus and SNc when motor signs appear, later on extends to the basal forebrain, amygdala, and the medial temporal lobe structures, and finally affects the convexity cortical areas in final stages [36]. Although this staging scheme is attractive since it fits well with the occurrence of nonmotor symptoms and signs across the clinical course of PD, it has been debated because it is based on autopsy and not on longitudinal studies, and it does not always hold true in all the patients [37]. In addition to a number of brain areas, neuronal loss and *α*-synuclein deposition involve also the peripheral nervous system, suggesting that PD is a multiorgan disease process, not merely a disorder of the central nervous system [38].

It has become increasingly evident that PD is a heterogeneous disorder in terms of symptoms and signs and natural history, and, based on cluster analysis, two PD subtypes have been proposed, namely, tremor-dominant PD and postural instability and gait difficulty (PIGD) PD [39, 40]. Tremor-dominant PD occurs earlier (20–40 years); it is often genetic and has good prognosis with slow progression, good response to levodopa, and motor fluctuations [40]. At variance, PIGD PD occurs sporadically after the age of 60 years with predominant bradykinesia and rigidity and earlier occurrence of depression and dementia [40]. Some studies suggested that the two PD subtypes show neuropathological differences, which include greater neuronal loss in the SNc, especially in its lateral portion, and the locus coeruleus, more severe dopamine loss in the ventral GPi and a larger number of cortical Lewy bodies in PIGD PD, and more severe loss of neurons in the midbrain retrorubral A8 field in tremor-dominant PD [40]. Although these data suggest that PD subtypes have different neuropathology, they are based on small autopsy studies, with no available biological markers that can lend support to this hypothesis in vivo [40].

## 4. The Neuropharmacology of PD

The neuronal loss and *α*-synuclein deposition in the SNc cause the involvement of dopaminergic neurons, the neuropharmacological hallmark of PD, and the rationale for the treatment with levodopa and dopamine agonists [35]. PD symptoms and signs appear only after substantial (i.e., >70%) degeneration of the SNc neurons, documenting remarkable compensatory phenomena within the nigrostriatal system [41].

The neuropathological changes in other brain areas result in degeneration of nondopaminergic pathways, which contributes to motor and nonmotor PD features. Nondopaminergic neurotransmitters and neuromediators include cholinergic, adenosinergic, glutamatergic, GABAergic, noradrenergic, serotonergic, opioidergic, and histaminergic systems (Table 2) [21]. The relative contribution of each of these pathways to single motor and nonmotor symptoms and signs and motor complications in PD is only partially explored, but they may represent potential targets for new pharmaceutical interventions [21]. In recent years, many RCTs have been completed and are ongoing or planned to explore drugs to counterbalance the loss of these neurotransmitters (Table 2), and it has been hypothesized that multiple targeting may be a more efficacious strategy, especially if they act in a synergistic manner [21].

Loss of cholinergic neurons in the PPN and the nucleus basalis of Meynert may contribute to posture and gait signs and falls through failure in the direct control of spinal circuitries and the deficits in the attentional processes required for these tasks [42, 43] and to cognitive impairment [7, 43]. The large aspiny interneurons in the striatum contain also large quantities of acetylcholine, which interacts with muscarinic and nicotinic receptors [44]. Centrally acting cholinesterase inhibitors, such as donepezil and rivastigmine, which delay acetylcholine degradation and prolong its effect, are commonly used in AD- and PD-related dementia and appear to offer promising preliminary results for gait disturbances in PD (Table 2) [21].

Adenosine acts with the A_2A_ receptors, which are located in the dendritic spines of the medium spiny neurons of the striatum and counteract the inhibitory action of indirect dopaminergic D2 receptors with no effect on the excitatory D1 pathway [21]. Drugs that antagonize A_2A_ receptor activity in combination with levodopa have been found to reduce off time in PD patients with motor fluctuations and improve on time with dyskinesia without changing the amount of troublesome peak-dose dyskinesia [45]. Caffeinated coffee consumption is inversely related to PD risk, suggesting a possible and largely debated neuroprotective effect of caffeine and/or effect on motor function [21]. Caffeine is a nonselective A_2A_ receptor antagonist that has been found to improve motor signs in PD in small trials [46], but larger RCTs are needed to confirm these preliminary findings and to establish whether they are sustained [21].

## 5. Pathophysiology of Bradykinesia in PD

The terms bradykinesia, hypokinesia, and akinesia collectively define a group of functional disturbance of voluntary movement prominently characterized by slowness [47]. Bradykinesia refers to slowness of movement that is ongoing, akinesia indicates failure of voluntary, spontaneous (e.g., in facial expression), or associated movement (e.g., arm swing during walking) to occur, and hypokinesia refers to movements that are smaller than desired, in particular with repetitive movements [47]. In addition to whole-body slowness, bradykinesia may impair the fine motor movements, which is usually demonstrated in PD patients during rapid alternating movements of fingers, hand, or feet as a progressive reduction of speed and motion amplitude [47]. Bradykinesia is represented cranially by loss of facial expression (hypomimia), decreased frequency of blinking, monotonic and hypophonic speech, and drooling due to decreased spontaneous swallowing. Other manifestations of bradykinesia are slowness in raising from a chair, loss of spontaneous gesturing, reduction of handwriting (micrographia), reduced arm swing when walking, and reduced gait amplitude and velocity [47]. Although both speed and movement amplitude are affected in PD, the former is usually disproportionally more affected in off state and less normalized by levodopa than the latter, suggesting that they may be associated with partially separate mechanisms [48].

The pathophysiology of bradykinesia is not completely understood, but among PD cardinal signs, it is the one that fits better with the classical model of the prevalence of the indirect pathway over the direct one in the BG (Figure 1) [14–16]. According to this model, failure of the BG output to reinforce the cortical mechanisms may involve the preparation of the movement or its execution [14–16, 47].

Deficits in movement preparation in PD patients have been documented by slower reaction times [49, 50] and slower increase in premovement cortical excitability [51], which together suggest abnormal retrieval of stored motor commands [47]. EEG studies showed premovement potential abnormalities [52], which were more marked in self-paced versus externally triggered movements [53] and are consistent with reduced activity in the supplementary motor area during movement programming [47]. EEG activity is physiologically represented by predominant alpha (10 Hz) and beta (20–30 Hz) range during motor inactivity and tonic position holding and when stopping a preplanned movement, while alpha and beta power is decreased ~1 s before movement [54]. Beta activity has been hypothesized to represent an idle rhythm that favors the status quo over new movements [55]. Premovement EEG beta desynchronization is reduced in PD patients, and this abnormality is at least partially normalized by dopaminergic stimulation [56]. Local field potential recording in the BG indicates a coupling between cortical and STN and GPi beta rhythms off medication, while they are decoupled on medication [57]. Beta band power suppression to levodopa was demonstrated to correlate with improvement in bradykinesia and rigidity but not tremor, suggesting a specific pathogenetic significance [58]. In accordance with these pieces of evidence, closed loop STN DBS, where stimulation frequency is automatically adjusted online according to the current state of the underlying network activity, may offer advantages over current fixed frequency (usually 130 Hz) DBS and its application could represent a therapeutic advancement in PD [59].

Deficits in movement execution include difficulties in producing maximal voluntary contraction [60] and abnormalities in the ballistic movement triphasic electromyographic pattern, which is composed of a first agonist muscle burst, followed by a second antagonist muscle burst and variably by a third agonist burst [47]. The size and duration of the first agonist burst in PD patients are reduced and suggest inappropriate scaling of the dynamic muscle force to the required movement parameters [61]. PD patients have additional difficulties represented by fatigue in complex or repetitive movements, and this can be clinically assessed when testing repetitive hand opening/closing or finger tapping [62]. It has also been suggested that PD patients have limited processing mechanisms that may interfere with their ability to run complex or simultaneous tasks [63].

Abnormalities of cortical excitability [64, 65], somatosensory function [66], and sensorimotor integration [67, 68] and changes in the pattern of activation in the motor and premotor cortices and the supplementary motor area [69, 70] may also contribute to deficits in movement execution in PD [47]. Whether these alterations represent true pathogenetic mechanisms of bradykinesia or compensatory changes is still unclear [64, 70, 71].

Secondary factors that may contribute to bradykinesia include muscle weakness [60], rigidity [72], rest and action tremor [73], and movement variability and bradyphrenia [47].

## 6. Pathophysiology of Tremor in PD

PD patients can show different tremor types [74, 75]. They include rest tremor, which stands among the PD cardinal signs, especially in the tremor-dominant subtype [4, 40], an action tremor named* reemergent tremor*, which reappears few seconds after the transition from rest to posture and has a frequency similar to that of rest tremor, essential tremor, dystonic tremor [74], and exaggerated physiological tremor [75]. We will focus on the pathophysiology of rest tremor, which is usually asymmetric with moderate amplitude, medium (4–6 Hz) frequency, and an agonist-antagonist alternate contraction pattern [76]. It typically involves the hand, manifesting as a pill-rolling movement, and less frequently the forearm as a pronation-supination, the leg as an adduction-abduction, the jaw, and/or head as a yes-yes or no-no motion [76]. Rest tremor is usually enhanced by motor or cognitive tasks and not influenced by weighting [76].

The pathophysiology of rest tremor is largely unknown, but there is good evidence that it differs from that of bradykinesia and rigidity [77]. Rest tremor can be more severe on the side opposite that of worse bradykinesia and the magnitude of tremor is not related to dopamine deficiency and does not respond readily to dopaminergic treatment [75]. Some reports suggest a role of dopaminergic loss in the midbrain retrorubral A8 field, which projects to the pallidum and is separate from the nigrostriatal pathways, in the genesis of rest tremor [32, 77]. The severity of rest tremor was found to correlate with a decrease in median raphe serotonin receptor binding [78], suggesting that serotoninergic rather than dopaminergic neuron loss might be more relevant to the pathogenesis of this symptom, but this point is controversial because serotoninergic drugs do not usually improve tremor in PD [75].

Several hypotheses, which share the view of a central rather than peripheral origin, have been suggested to explain the pathophysiology of rest tremor [32]. Bursts that are correlated with tremor have been demonstrated in a number of cortical and subcortical areas, but the exact localization of the primary tremor pacemaker is still debated [32, 76]. Thalamocortical relay neurons have ion channel properties that support pacemaking at approximately rest tremor frequency and may be modulated through hyperpolarization by reducing excitatory drive or excitatory input from the cerebellum [79]. Other models suggest a role of the recurrent loop between GPe and the STN as the primary oscillator [80] and the STN-cortical oscillatory coupling [81]. The cerebellum seems to have a central role in PD tremor pathogenesis, because rest tremor disappears following lesions of the ventralis intermedius (VIM) thalamic nucleus, which receives cerebellar input, and cerebellar stimulation may alter the timing of peripheral tremor. An emergent model indicates abnormally synchronized BG-thalamocortical (BGTC) loop, a GPe-STN pacemaker, and the cerebellar dentate-thalamocortical (CTC) circuit as the main actors producing rest tremor [32, 77]. According to this hypothesis, the GPe-STN pacemaker and the BGTC loop trigger tremor episodes, and the CTC circuit maintains and modulates their amplitude [77]. This model is in accordance with the observation that stereotactic lesions in selected areas of the BGTC (STN, primary motor cortex, ventrolateral thalamic nucleus, and pallidum) or the CTC (VIM) may abolish rest tremor [77, 81].

## 7. Pathophysiology of Rigidity in PD

PD rigidity is characterized by increased muscle tone to palpation at rest, reduced distension to passive movement, increased resistance to stretching, and facilitation of the shortening reaction [82]. Rigidity is more marked in flexor than extensor muscles, may be enhanced by voluntary movement of other body parts, and is more remarkable during slow than fast stretching, and these features help differentiating PD rigidity from spasticity, which is worse during fast displacement [82, 83]. Cogwheel phenomenon is the result of coexisting rigidity and tremor [82].

The pathogenesis of PD rigidity has been hypothesized to include changes in the passive mechanical properties of joints, tendons, and muscles, the enhancement of stretch-evoked reflexes from segmental spinal or supraspinal activity, and abnormalities in peripheral sensory inputs that may influence the response to muscle stretch [83–86]. Studies on spinal reflexes indicate a shift of spinal cord motoneurons towards increased activity in response to peripheral stimulation [84, 85] and increased response to muscle stretch [83], with a possible contribution of transcortical long-latency stretch reflex [86]. How these changes are associated with dopamine deficiency and BG output abnormalities, which are stipulated by the classical BG pathophysiological model, is still unclear [82].

## 8. Pathophysiology of Motor Fluctuations and Dyskinesia

After several years of smooth and stable response to oral levodopa treatment, PD patients invariably develop motor complications, which include motor fluctuations and dyskinesia [87, 88]. Motor fluctuations include wearing-off, delayed-on, partial-on, no-on, and on-off fluctuations (Table 3) [87]. Dyskinesia is choreic, ballistic, or dystonic involuntary movements and can be classified into peak-dose, diphasic, and square-wave dyskinesia (Table 3) [87]. Dystonia often accompanies motor fluctuations and dyskinesia and may appear in off and on phases (Table 3) [87].

The pathogenesis of motor complications is not completely understood, but central and peripheral mechanisms have been suggested to contribute to motor fluctuations and dyskinesia [88]. The main central mechanisms include (a) the progression of nigrostriatal degeneration, which results in the reduction of the capacity of storing dopamine in the presynaptic vesicles and releasing them physiologically, (b) enhanced conversion of levodopa to dopamine and aberrant release in the striatum as false neurotransmitter by serotoninergic neurons, (c) alterations in dopaminergic receptors that undergo plastic changes, which include supersensitivity to dopamine because of the loss of nigrostriatal projections, desensitization, and downregulation because of the presence of nonphysiological high doses of dopamine, and (d) increased glutamatergic activity in the striatum [87, 88]. The peripheral mechanisms encompass (a) reduced gastric emptying that is related to PD autonomic dysfunction and (b) competition of levodopa, which is a neutral aminoacid and requires a carrier to pass the gut-blood and blood-brain barriers, with other dietary amino acids after a protein-rich meal [87]. The cumulative exposure to levodopa treatment, which becomes necessary after a few years of PD disease because of the limited therapeutic effect of dopamine agonists, has been traditionally considered as a major player in the pathogenesis of motor fluctuations and dyskinesia, which are called levodopa-induced motor complications [87, 88]. A recent study on PD patients from a sub-Saharan African country, where access to medication is limited, suggests that motor complications are not associated with the duration of levodopa therapy but rather with longer disease duration and higher levodopa daily dose, arguing against the common practice to delay levodopa treatment in favor of dopamine agonists to delay the occurrence of motor complications [89].

Management strategies for motor complications and dyskinesia include various pharmacological combined approaches, such as fractionating levodopa by administering small multiple daily doses, reducing the interval between levodopa doses, adding controlled release, dispersible, and soluble levodopa formulations, adding or increasing dopamine agonists in particular controlled release and transdermal formulations, monoamine oxidase-B inhibitors or catechol-O-methyltransferase inhibitors, amantadine or clozapine, botulinum toxin, subcutaneous apomorphine, levodopa/carbidopa intestinal gel, and DBS (Table 3) [87]. Other strategies include adjusting protein intake throughout the day, taking levodopa on an empty stomach, treating constipation, tapering drugs that may interfere with gastric emptying, and eradicating* Helicobacter pylori* (Table 3) [87].

## 9. Posture, Balance, and Gait Disturbances in PD and Their Pathophysiology

Posture, balance, and gait disturbances are common in PD and largely contribute to motor impairment, risk of falls, and worse QoL [90, 91]. PD patients commonly show the classic stooped appearance, with flexion of the hips and knees, and rounding of the shoulders, but an important subset of patients shows more severe postural deformities, including camptocormia, antecollis, Pisa syndrome, and scoliosis [91, 92]. The pathophysiology of axial postural abnormalities in PD is not well understood, and a number of central and peripheral causes have been proposed, including asymmetry of the BG outflow, rigidity, dystonia, abnormal processing of vestibular or proprioceptive afferents, abnormal spatial cognition, focal myopathy in the paraspinal muscles, spinal and soft tissue changes, and side effects of dopaminergic and nondopaminergic drugs [90, 92]. Because of the poor knowledge on the pathogenesis of postural abnormalities, their management is largely unsatisfactory, as they respond poorly to medication, brain surgery, or physiotherapy [92].

Gait and balance disorders, which occur during the course of PD, are a major problem and an unmet therapeutic target, in that dopaminergic drugs and DBS often fail to improve these signs and may worsen them in some cases [91]. Gait is the result of dynamic interactions between the activation of central movement programs and feedback mechanisms [93]. Animal studies demonstrated the presence of a spinal central pattern generator (CPG), which is controlled by supraspinal centers [93]. Recent studies point to a key role of the mesencephalic locomotor region (MLR), which is located in the reticular formation and is composed of the PPN and the cuneiform nucleus, for the control of gait and balance in humans [93, 94]. The MLR has reciprocal connections with the BG, receives inputs from the cerebellum and motor cortices, and has outputs to the descending reticulospinal pathway and the ascending thalamocortical pathway through the thalamic centromedian nucleus [91]. The spinal CPG and the MLR are under cortical control [93]. An indirect pathway from the frontal cortex via the BG to the MLR allows modulation of the gait pattern in response to external demands [95]. A direct pathway from the primary motor cortex to the spinal CPG can bypass the MLR during undisturbed locomotion [95]. Input from the cerebellum conveys both pathways in the MLR to control speed and gait pattern, according to proprioceptive, vestibular, and visual information [93, 95]. Given the complex anatomy underlying locomotion, gait and balance signs may be heterogeneous in PD patients [91]. In early PD, hypokinetic gait, which is characterized by reduced gait speed and amplitude with nearly normal cadence, is an expression of the bradykinesia and is related to a deficit in internal generation of adapted step length [96]. In line with this concept, hypokinetic gait may be ameliorated by dopaminergic treatment and external visual cues [91]. The characterization of gait problems is more complex in later PD stages, when changes in multiple neural systems may contribute to them [21, 91, 97]. In advanced PD patients, gait is clearly abnormal, but it is often difficult to distinguish between the specific contribution of sensory, motor, and cognitive (i.e., executive functions and attention) deficits and factors like fear, imbalance, muscle weakness, loss of basic locomotion rhythmicity, and misjudgment of hazard risk [97]. In later PD, different neurotransmitters have been hypothesized to contribute to gait disturbance, including noradrenalin and serotonin systems, but converging evidence points to a key role of cholinergic dysfunction in the PPN [21, 42, 43, 94]. According to this view, some reports showed improvement of PD gait disturbances to inhibitors of cholinesterase [21, 43]. Despite these results needing to be replicated in more robust studies, this pharmacological strategy should not be discarded [91]. Bilateral PPN low frequency DBS was suggested to effectively control gait and balance disorders in small samples of patients, but these preliminary data were not confirmed by two RCTs [98], suggesting that better criteria for selecting patients and for optimal targeting within the MLR are needed [91].

## 10. Pathophysiology of Freezing of Gait

Freezing of gait (FOG) is an episodic gait disturbance [97], which is characterized by difficulty in gait initiation (*start hesitation*) and paroxysmal unintentional episodes of motor block during walking [99]. A FOG episode can manifest with step size reduction (*shuffling gait*), knee trembling, or akinesia and is typically described as feeling the feet as* frozen* or* glued to the ground *[100]. FOG is often triggered or worsened by challenging situations or provocative environments, such as changing direction (*turning hesitation*), approaching narrow doorways (*tight quarter hesitation*) or destinations (*destination hesitation*), moving into crowded spaces, walking on a slippery surface, crossing thresholds or changes in floor, stepping into an elevator, or entering a revolving door [100]. Furthermore, freezing episodes can occur when patients are required to deal with simultaneous activities (*dual tasking*), like walking and talking [99, 101]. Emotional factors such as stress or anxiety may also contribute to triggering FOG episodes [100]. All the above circumstances require a dynamic adaptation of motor schema, because of an increased cognitive load [100, 101]. Different subtypes of FOG are defined according to clinical manifestations and response to external stimuli (e.g., visual or auditory cues) and to levodopa [100]. It has long been observed that freezing phenomena in PD patients are responsive to visual cues, such as stepping over a small obstacle (e.g., a foot or a laser on the cane/walker), or auditory cues, such as following a rhythm (e.g., counting, listening to a metronome or music) to step to the beat, and this clinical observation offers a rationale for some rehabilitation strategies for FOG [91]. FOG is common in advanced PD and is associated with increased risk of falls and reduced mobility and QoL [102].

The neuropharmacological bases of FOG are poorly understood [99, 100]. Despite being common in advanced PD patients, FOG may appear early in the disease course [100]. Moreover, the response of FOG to dopaminergic therapy and DBS may be poor, and this clinical phenomenon is not unique to PD [100]. These observations suggest that severe dopamine depletion alone could not explain FOG and critical brain regions for this phenomenon should differ from those involved in cardinal PD features [94, 100]. As for other gait disturbances in PD, cholinergic loss in the PPN, which stands at the crossroad between supraspinal and spinal gait centers, may play a role in FOG [93, 94]. In keeping with this hypothesis, bilateral DBS of the caudal PPN may improve FOG [103]. Cortical cholinergic loss and amyloid deposition [104] and gray matter atrophy in the inferior parietal lobe and angular gyrus [105] may also contribute to FOG pathophysiology.

There are several theories regarding the occurrence of FOG [100, 106]. Based on the association between its occurrence and some visual stimuli, such as passing through a narrow space, FOG has been suggested to depend on impaired visuospatial ability that interferes with online movement planning [106, 107]. Visuospatial tests may discriminate freezers from nonfreezers and their deficits are strongly related with FOG severity and metrics [108], but other studies contradicted the notion that the lack of visuospatial ability per se may be primarily responsible for FOG [107, 109].

Impaired coupling between postural control and step initiation has also been hypothesized to contribute to FOG, because of its strong correlation with postural instability [100]. While a single anticipatory postural correction that shifts weight off the stepping leg precedes a voluntary step in normal gait, PD patients with FOG show delayed step initiation associated with repetitive anticipatory postural adjustments, as if they cannot inhibit their postural preparation and release the stepping program [110].

The hypotheses on FOG pathophysiology have recently shifted towards a multisystem dysfunction, where cognition plays a significant role [101, 111]. Although gait has been long considered a low-level automated motor activity that requires minimal higher cortical functions, growing evidence suggests a role for cognition, especially attention and executive functions in gait control [99]. Different models, which incorporate cognition in the pathogenesis of FOG, have been recently proposed and some of them will be briefly reviewed [111].

According to the* interference model*, FOG is the consequence of concurrent cognitive overload during walking [112]. This model suggests that reduced neural reserve in the BG leads to communication breakdown between motor, associative, and limbic parallel circuits causing abnormal pallidal output and temporary interruption of gait pathways [112]. Because of reduced automaticity in PD, there is an overload of systems involved in performing voluntary actions, especially when patients are asked to perform a dual task [99, 101]. According to this hypothesis, during a virtual reality gait task where the cognitive load was manipulated, PD patients with FOG demonstrated functional decoupling between the BG and the cognitive control networks in association with the occurrence of paroxysmal motor arrests [113].

The* cognitive model* stipulates that impaired decision making because of executive dysfunction [114] leads to stronger automatic activation of incorrect responses and less efficient suppression of conflicting responses and results in delayed response selection and FOG [111]. Executive functions are an umbrella term for a set of abilities, which are involved in inhibition, switching, and updating, and flexibly control behavior towards goals [115, 116]. Among executive functions, difficulties in set shifting have stronger association with the presence of FOG [113]. The frontostriatal circuits are central for action selection and response inhibition, in signaling conflict and temporarily preventing premature action by raising the decision threshold, such that response selection is delayed until conflict is resolved [117]. A growing body of evidence suggests that cognitive impairment, in particular executive dysfunction, often coexists with posture and gait abnormalities, FOG, and risk of falls in PD, but their interplay appears to be complex [118] and may represent a promising basis for new rehabilitative approaches to treat gait disturbances in PD patients [99].

## 11. Fatigue and Pain in PD

Fatigue is defined as an overwhelming sense of tiredness, lack of energy, and feeling of exhaustion, with difficulties in initiating and sustaining mental and physical tasks in the absence of motor or physical impairment and consisting of a mental and physical component [119, 120]. The pathophysiology of PD-related fatigue appears to be complex, in that it involves both motor and nonmotor mechanisms, which depend on the involvement of nondopaminergic and extrastriatal dopaminergic pathways [119].

PD patients often complain of pain, which may be associated or not with the presence of dystonia in the same body regions affected by pain [121]. Experimental evidence suggests the presence of abnormal processing of nociceptive afferents in pain pathways, independently of dystonia and motor disturbances in PD [122, 123].

Despite not representing motor disturbances, fatigue and pain may negatively influence motor performances in PD [120]. Some reports suggested that caffeine [46] and monoamine oxidases inhibitors alone or in combination with antidepressants [124] may improve fatigue in PD and that opioids might be effective for some subtypes of PD-related pain [125]. Despite these recent advancements, pain and fatigue are two symptoms that are underrecognized and with no established therapy in PD, and they may represent interesting targets for nonpharmacological treatments, such as aerobic exercise [126] or rehabilitation procedures.

## 12. Depression, Apathy, and Cognitive Problems in PD

Depression, apathy, and cognitive deficits are common in PD patients and may sometimes overlap and interact [127]. Despite the fact that they can have a negative effect on QoL and the functioning of PD patients, as well as reduced compliance to pharmacological and nonpharmacological treatment, they are often underrecognized and undertreated [127, 128].

Depression may affect 50–70% of PD patients, is a multifactorial condition, which depends upon degeneration of noradrenaline and serotonin neurons, and represents a reactive condition to PD [129]. Pharmacologic treatment with antidepressant medications, specifically the selective serotonin reuptake inhibitors, and cognitive behavioral interventions may significantly improve depression in PD patients [129].

Apathy is defined as lack of motivation characterized by diminished goal-oriented behavior and cognition and reduced emotional expression [26, 128]. Although apathy can occur as a symptom of depression, it may represent a separate phenomenon in PD [130]. While depression is a highly negative affective experience, apathy is characterized by complete affective flattening in the absence of sadness [26]. However, in the clinical setting, separating depression from apathy is often not a straightforward task [26, 128]. Up to 40% of PD patients suffer from apathy, which is more common in older men with more severe motor impairment, worse executive dysfunction, and a higher risk of developing dementia [130]. Apathy in PD is caused by a dysfunction or neuronal loss in a complex neural network, which is not limited to the limbic loop of the BG, but includes the mesocorticolimbic pathway, the caudate nucleus, the lateral prefrontal cortices, the inferior medial frontal gyrus, the cingulate cortex, the insula, the cuneus, and the temporoparietal region [131, 132]. The treatment of apathy in PD is currently controversial, but there is a good rationale for the use of dopaminergic drugs to improve the emotional and behavioral aspects and for cholinesterase inhibitors to treat the cognitive aspects of apathy [26, 133].

Cognitive deficits may affect every cognitive domain, including memory, language, attention, visuospatial abilities, and executive functions, with the latter showing the most profound impairment [134]. The spectrum of cognitive dysfunction in PD ranges from mild cognitive impairment (MCI) to dementia, with MCI representing a transitional state between normal cognition and dementia [7, 135] The recent introduction of diagnostic criteria for PD-related MCI [135] is important for its early recognition and treatment [7].

## 13. Rehabilitation Procedures in PD and Their Pathophysiological Grounds

Despite optimal medical treatment and neurosurgical interventions, PD patients develop progressive disability [136]. The role of rehabilitation in PD is to maximize motor and cognitive functional abilities and minimize secondary complications in order to optimize independence, safety, and well-being, thus enhancing QoL [137]. Several rehabilitative approaches have been proposed in PD, including nonspecific physiotherapy (i.e., muscle strengthening and stretching, balance, and postural exercises) [138, 139], occupational therapy [140], treadmill and robotic training [141–145], dance and martial arts therapy [146], multidisciplinary approaches including speech and cognitive therapy [8, 147, 148], motor imagery and action observation therapy [137, 149], and virtual reality and telerehabilitation [150]. There is evidence that physiotherapy causes short-term, significant, and clinically important benefit for walking speed, balance, and clinician-rated disability in PD [138], but it is insufficient to support or refute the superiority of an intervention over another because of the small number of patients examined by previous studies, the methodological flaws, and the variety of the approaches that have been proposed [137]. However, exercise is generally accepted as an intervention that could ameliorate motor and nonmotor PD symptoms and should be considered as the basic element of any rehabilitative treatment in PD patients [126, 137].

The principles of neuromechanics are a framework for understanding the patterns of neural activity that generate movements in healthy people and are important for the rehabilitation of patients with motor deficits [151]. Together with neural plasticity, they support the development of motor modules, which have been defined as coordinated patterns of muscle activity flexibly combined to produce functional motor behaviors [151]. The neuromechanical principles include motor abundance (i.e., for any given task, there are many functionally equivalent motor solutions), motor structure (i.e., motor modules reflect biomechanical task relevance), motor variability (i.e., motor module variations across individuals are high if the effect on motor output is low), individuality (i.e., individuals express different motor styles that depend on evolutionary, developmental, and learning processes), and multifunctionality (i.e., muscle activity can be combined in many ways to produce a wide range of different actions) [151].

BG loops have been hypothesized to contribute to choosing the desired motor output and selectively inhibiting competing motor programs [152] and to be involved in reward prediction and habit formation [153, 154]. In PD, the BG dysfunction is supposed to lead to inappropriate selection of motor modules [151, 152]. Upon these premises, PD rehabilitation procedures are aimed to improve the appropriate recruitment of motor modules through exercise and practice of complex tasks according to a goal-based learning approach, which involves planning and execution of composite and/or unfamiliar movements (e.g., backward walking) [137, 151].

Motor rehabilitation may be regarded as a process of motor relearning through practice and training [155]. The acquisition of motor skills is supposed to go through different phases (i.e., fast, slow, consolidation, reconsolidation, automatization, and retention), which differentially involve the corticostriatal and corticocerebellar pathways and depend upon online and offline triggered plastic changes in the brain [156]. PD patients show preserved ability in motor learning [155], but BG dysfunction may impair consolidation of learned material, and translation to the clinical setting may be critical [137]. Along this line, reduced experience-dependent neuroplasticity, which is largely influenced by intensity, repetition, specificity, difficulty, and complexity of practice, may represent a crucial issue in PD [137]. Motor cortical plasticity may be a compensatory change that contributes to delaying motor signs onset in the early phases of PD, but it deteriorates as the disease progresses [157].

Other largely unexplored mechanisms involved in PD rehabilitation include focusing on external cues to bypass the dysfunctional BG activity and access the corticocerebellar pathways [158], enhancing cognitive engagement through problem solving, attentional demand and motivation [137], and aerobic training to increase cardiopulmonary function, oxygen consumption, and blood flow to the brain [159].

## 14. Conclusions and Future Perspectives

This brief review summarized the current hypotheses on the pathophysiology of motor dysfunction in PD. The neuropathological, neurochemical, and neurophysiological bases of PD motor symptoms offer the rationale for current pharmacological and nonpharmacological treatment of this condition but may also represent the bases for future strategies for managing this condition [21, 137]. Future studies aimed at a better understanding of PD pathophysiology will offer the premises for new pharmacological strategies [21], as well as new targets for DBS [59, 87, 98] and rehabilitation procedures [160], and to achieve a personalized medicine approach to PD based on biomarkers [161].

## Figures and Tables

**Table 1:** The glossary of the main motor and nonmotor symptoms and signs in Parkinson's disease.

Symptom or sign	Description
Cardinal motor symptoms and signs	
Bradykinesia	Slowness of voluntary movement and/or a movement that is ongoing. For the companion terms akinesia and hypokinesia, see below
Rest tremor	Asymmetric 4–6 Hz moderate amplitude tremor, which usually involves the thumb (*pill-rolling* tremor). It may involve other body parts, such as the forearm pronation/supination, the leg adduction/abduction, and the jaw. Head tremor is rarely seen in PD. For other PD tremor types, see the text
Rigidity	Increased muscle tone felt during examination by passive movement of the affected segment, involving both flexor and extensor muscles and not increased with higher mobilization speed (in contrast with spasticity)
Postural instability	Impaired postural adjustment due to decrease or loss of postural reflexes
Other motor symptoms and signs (early and advanced disease stages)	
Akinesia	Reduction, delay, or absence of either voluntary, spontaneous, or associated movement
Hypokinesia	Reduced movement amplitude, particularly with repetitive movements
Hypomimia	Reduced facial expression
Hypophonia	Reduced voice volume
Micrographia	Small handwriting that becomes progressively smaller and less readable
Festination	Involuntary gait acceleration with step shortening
Tachyphemia	Acceleration of speech segments
Sialorrhea	Drooling of saliva
Dysarthria	Slurred speech
Dysphagia	Difficulty in swallowing
On phase	A phase characterized by a beneficial effect of levodopa with release from the parkinsonian symptoms and signs
Off phase	A phase, in which the parkinsonian symptoms and signs take over, sometimes in the form of a crisis with severe bradykinesia, rigidity, and tremor. Nonmotor off features include pain, paresthesia, sweating, thoracic oppression, and anxiety symptoms
Freezing of gait	Difficulty in gait initiation (start hesitation) and paroxysmal unintentional episodes of motor block during walking
Postural instability	Impaired postural adjustment due to decrease or loss of postural reflexes
Akathisia	Feeling of inner restlessness and strong need to be in constant motion associated with the inability to sit or stay still
Camptocormia	Abnormal involuntary flexion of the trunk that appears when standing or walking and disappears in the supine position
Anterocollis	Marked neck flexion (>45%), disproportionate to trunk flexion
Pisa syndrome	Tonic lateral flexion of the trunk associated with slight rotation along the sagittal plane
Selected nonmotor symptoms and signs	
Hyposmia/anosmia	Reduction/loss of the sense of smell
Constipation	Infrequent and frequently incomplete bowel movements
Orthostatic hypotension	A decrease in systolic blood pressure of at least 20 mm Hg or a decrease in diastolic blood pressure of at least 10 mm Hg within three minutes of standing when compared with blood pressure from the sitting or supine position
Fatigue	Overwhelming sense of tiredness and feeling of exhaustion with difficulties in initiating and sustaining mental and physical tasks
Apathy	Lack of motivation characterized by diminished goal-oriented behavior and cognition and reduced emotional expression
Restless legs syndrome	Movement disorder characterized by compelling urge to move the legs, particularly when in bed and trying to sleep

For depression, cognitive problems, and pain, see the text, PD: Parkinson's disease.

**Table 2:** Nondopaminergic neurotransmitters involved in the pathogenesis of Parkinson's disease and pharmacological agents potentially active or tested to counteract their deficit.

Neurotransmitter	Site	Symptom/sign	Drug
Acetylcholine	PPN, nucleus basalis of Meynert, striatum	Posture and gait disturbances, FOG, cognitive problems	Cholinesterase inhibitors, nicotinic receptor agonists
Adenosine	Striatum	Motor fluctuations, dyskinesia	Adenosine A_2A_ receptor antagonists, caffeine
GABA	GPe, STN	Motor fluctuations, dyskinesia	GAD gene therapy
Glutamate	Striatum, STN	Dyskinesia, FOG	NMDA receptor antagonists, AMPA receptor antagonists, mGluNAMs
Histamine	Striatum	Dyskinesia	H_2_ receptor antagonists
Noradrenaline	GPe, locus coeruleus	Balance and gait disturbances, FOG, dyskinesia	Methylphenidate, *α*_2_ receptor antagonists
Serotonin	Dorsal raphe nucleus, striatum, GP, SN	Motor fluctuations, dyskinesia	5-HT_1A_ receptor antagonists

5-HT_1A_: serotonin receptor 1A; A_2A_: adenosine receptor A2; AMPA: alpha-amino-3-hydroxy-5-methyl-4-isoxazolepropionic acid; FOG: freezing of gait; GABA: gamma-aminobutyric acid; GAD: glutamic acid decarboxylase; GP: globus pallidus; GPe: external segment of the globus pallidus; mGluNAMs: metabotropic glutamate receptor negative allosteric modulators; NMDA: N-methyl-D-aspartate; PPN: pedunculopontine nucleus; SN: substantia nigra; STN: subthalamic nucleus.

**Table 3:** Levodopa (LD)-induced motor fluctuations and dyskinesia, their pathophysiology, and treatment strategies.

Phenomenon	Description	Pathophysiology	Treatment strategies
Motor fluctuations			
Wearing-off	Predictable earlier end-of-dose deterioration and reemergence of PD motor/nonmotor symptoms/signs before the next scheduled oral LD dose	Loss of SNc dopaminergic neurons resulting in reduction in LD internalization and production, storage, and physiological release of DA	Assess compliance with current treatment. Reduce the interval between LD doses. Increase LD doses, particularly the first one in the morning or those in the afternoon. Use CR-LD. Add or increase DA agonists. Add COMT inhibitors and/or MAO-B inhibitors. Consider SA, LCIG, or DBS
Delayed-on	Increased latency between taking an oral dose of LD and experiencing clinical benefit from it	Delayed absorption of LD in the proximal jejunum or across BBB because of large amount of dietary neutral AAs that compete with LD active transport, erratic gastric emptying, anticholinergic or dopaminergic drugs, and food *per se*	Adjust protein intake by avoiding it in the first part of the day or spreading it throughout the day. Take LD on an empty stomach or with a small snack. Treat constipation and reduce or stop anticholinergic agents. Eradicate *Helicobacter pylori*. Add soluble oral LD preparations. Consider SA, LCIG, or DBS
Partial-on	Partial response to an oral dose of LD	Reduced absorption of LD. *See pathophysiology of delayed-on fluctuations*	*See treatment strategies for delayed-on fluctuations*
No-on	Occasionally no response of PD symptoms/signs to an oral dose of LD	*See pathophysiology of delayed-on fluctuations. *Markedly reduced or absent absorption of LD	*See treatment strategies for delayed-on fluctuations*
On-off	Sudden and unpredictable fluctuations between on and off phases (Table 1)	Possible pharmacodynamic neuroplastic changes in striatal medium spiny neurons and the BG	*See treatment strategies for wearing-off fluctuations*
Dyskinesia			
Peak-dose dyskinesia	Involuntary movements at the time of the LD peak, which coincide with the best antiparkinsonian effect of LD	Loss of SNc dopaminergic neurons resulting in reduction in LD internalization and leading to greater amount of DA production by serotoninergic neurons. Neuroplastic changes in DA and GABA receptors and overactivity of glutamatergic NMDA receptors in the BG. Disinhibition of the MC and associated motor cortices	Fractionate LD doses (smaller amounts, more frequently). Switch CR-LD to regular LD. Add or increase long-acting DA agonists. Discontinue COMT or MAO-B inhibitors. Add amantadine or clozapine. Consider SA, LCIG, or DBS
Diphasic dyskinesia	Involuntary movements at the beginning and/or the end of LD effect	*See pathophysiology of peak-dose dyskinesia*	Reduce the interval between LD doses. Add or increase long-acting DA agonists. Add soluble or crushed oral LD. Consider SA, LCIG, or DBS
Square-wave dyskinesia	Involuntary movements throughout the entire duration of LD effect	*See pathophysiology of peak-dose dyskinesia*	*See treatment strategies for peak-dose and diphasic dyskinesia*
Dystonia			
Off phase dystonia, including early morning dystonia	Sustained involuntary and painful muscle contraction during the off phase and/or on awakening	*See pathophysiology of wearing-off and delayed-on fluctuations. *Short half-life of oral LD for early morning dystonia	*See treatment strategies for motor fluctuations. *Minimize off time. Add bedtime CR-LD or overnight doses of regular LD for early morning dystonia. Botulinum toxin injection. Add muscle relaxant drugs or benzodiazepines. Consider DBS
On phase dystonia	Sustained involuntary muscle contraction during the on phase. It may often accompany peak-dose or diphasic dyskinesia	*See pathophysiology of peak-dose dyskinesia*	*See treatment strategies for peak-dose dyskinesias. *Botulinum toxin injection. Add muscle relaxant drugs or benzodiazepines. Consider DBS

AA = aminoacid; BBB = blood-brain barrier; BG = basal ganglia; COMT = catechol-O-methyl transferase; CR = controlled release; DA = dopamine; DBS = deep brain stimulation; GABA = gamma-aminobutyric acid; LCIG = levodopa/carbidopa intestinal gel; LD = levodopa; MC = primary motor cortex; MAO-B = monoamine oxidase type B; NMDA: N-methyl-D-aspartate; PD = Parkinson's disease; SA = subcutaneous apomorphine.
